# Anosmia, ageusia, and other COVID-19-like symptoms in association with a positive SARS-CoV-2 test, across six national digital surveillance platforms: an observational study

**DOI:** 10.1016/S2589-7500(21)00115-1

**Published:** 2021-07-22

**Authors:** Carole H Sudre, Ayya Keshet, Mark S Graham, Amit D Joshi, Smadar Shilo, Hagai Rossman, Benjamin Murray, Erika Molteni, Kerstin Klaser, Liane D Canas, Michela Antonelli, Long H Nguyen, David A Drew, Marc Modat, Joan Capdevila Pujol, Sajaysurya Ganesh, Jonathan Wolf, Tomer Meir, Andrew T Chan, Claire J Steves, Tim D Spector, John S Brownstein, Eran Segal, Sebastien Ourselin, Christina M Astley

**Affiliations:** a School of Biomedical Engineering & Imaging Sciences, King's College London, London, UK; b Medical Research Council Unit for Lifelong health and Ageing at UCL, Department of Population Science and Experimental Medicine, University College London, London, UK; c Centre for Medical Image Computing, Department of Computer Science, University College London, London, UK; d Department of Computer Science and Applied Mathematics and Department of Molecular Cell Biology, Weizmann Institute of Science, Rehovot, Israel; e Clinical and Translational Epidemiology Unit and Division of Gastroenterology, Massachusetts General Hospital and Harvard Medical School, Boston, MA, USA; f Pediatric Diabetes Unit, Ruth Rappaport Children's Hospital, Rambam Healthcare Campus, Haifa, Israel; g Department of Twin Research and Genetic Epidemiology, King's College London, London, UK; h ZOE Global, London, UK; i Computational Epidemiology Lab, Boston Children's Hospital, Boston, MA, USA; j Division of Endocrinology, Boston Children's Hospital, Boston, MA, USA; k Broad Institute of Harvard and Massachusetts Institute of Technology, Cambridge, MA, USA; l AI Institute 3IA Côte d'Azur, Université Côte d'Azur, Nice, France

## Abstract

**Background:**

Multiple voluntary surveillance platforms were developed across the world in response to the COVID-19 pandemic, providing a real-time understanding of population-based COVID-19 epidemiology. During this time, testing criteria broadened and health-care policies matured. We aimed to test whether there were consistent associations of symptoms with SARS-CoV-2 test status across three surveillance platforms in three countries (two platforms per country), during periods of testing and policy changes.

**Methods:**

For this observational study, we used data of observations from three volunteer COVID-19 digital surveillance platforms (Carnegie Mellon University and University of Maryland Facebook COVID-19 Symptom Survey, ZOE COVID Symptom Study app, and the Corona Israel study) targeting communities in three countries (Israel, the UK, and the USA; two platforms per country). The study population included adult respondents (age 18–100 years at baseline) who were not health-care workers. We did logistic regression of self-reported symptoms on self-reported SARS-CoV-2 test status (positive or negative), adjusted for age and sex, in each of the study cohorts. We compared odds ratios (ORs) across platforms and countries, and we did meta-analyses assuming a random effects model. We also evaluated testing policy changes, COVID-19 incidence, and time scales of duration of symptoms and symptom-to-test time.

**Findings:**

Between April 1 and July 31, 2020, 514 459 tests from over 10 million respondents were recorded in the six surveillance platform datasets. Anosmia–ageusia was the strongest, most consistent symptom associated with a positive COVID-19 test (robust aggregated rank one, meta-analysed random effects OR 16·96, 95% CI 13·13–21·92). Fever (rank two, 6·45, 4·25–9·81), shortness of breath (rank three, 4·69, 3·14–7·01), and cough (rank four, 4·29, 3·13–5·88) were also highly associated with test positivity. The association of symptoms with test status varied by duration of illness, timing of the test, and broader test criteria, as well as over time, by country, and by platform.

**Interpretation:**

The strong association of anosmia–ageusia with self-reported positive SARS-CoV-2 test was consistently observed, supporting its validity as a reliable COVID-19 signal, regardless of the participatory surveillance platform, country, phase of illness, or testing policy. These findings show that associations between COVID-19 symptoms and test positivity ranked similarly in a wide range of scenarios. Anosmia, fever, and respiratory symptoms consistently had the strongest effect estimates and were the most appropriate empirical signals for symptom-based public health surveillance in areas with insufficient testing or benchmarking capacity. Collaborative syndromic surveillance could enhance real-time epidemiological investigations and public health utility globally.

**Funding:**

National Institutes of Health, National Institute for Health Research, Alzheimer's Society, Wellcome Trust, and Massachusetts Consortium on Pathogen Readiness.

## Introduction

Participatory syndromic surveillance has informed public health for nearly a decade,^1^, ^2^ although it was the COVID-19 pandemic that spurred the rapid development of multiple digital monitoring platforms^3^, ^4^, ^5^, ^6^, ^7^, ^8^, ^9^ to accelerate our understanding of and response to SARS-CoV-2 globally.^10^ These population science initiatives encompass various participant interfaces including websites,^3^, ^5^, ^9^ telephone calls,^5^ text messages,^9^ and smartphone apps,^4^, ^6^ using cross-sectional and longitudinal study designs and implementing varying degrees of wide-scale sampling or engagement.

Real-time, community-based data from these platforms are strongly complementary to the so-called hard outcomes—that is, COVID-19 cases, hospitalisations, and deaths^11^—particularly in the setting of inadequate testing, delayed or absent reporting, or when ascertained outcomes only capture the most severe cases (eg, clinical features of patients hospitalised with COVID-19).^12^, ^13^ As an example of the usefulness of such platforms, the prediction of COVID-19 infection with symptom-based scores was pioneered with use of data from these platforms in response to the insufficient testing capacity at the start of the pandemic, highlighting early on the potential importance of smell and taste disorders.^8^, ^14^

**Research in context**

**Evidence before this study**

We searched PubMed for titles and abstracts in English that included the words “COVID” and “symptoms” but excluding “long” or “post” published between Jan 1, 2020, and Oct 31, 2020. This search yielded 99 results. We repeated the search with a focus on surveys by including in addition the words “survey” or “digital platform”, which yielded 75 results. As the COVID-19 pandemic evolved, testing capacity was expanded and governmental guidelines adapted, generally encouraging testing with a broader set of symptoms beyond fever with canonical respiratory symptoms. In parallel, multiple large-scale, participatory, digital surveillance platforms launched to complement knowledge from laboratory and somewhat smaller clinical studies. Symptoms such as loss of smell (anosmia) have been identified as strongly predictive of COVID-19 infection in both clinical and syndromic surveillance analyses and have thus been used to inform these testing policy changes and access expansion.

**Added value of this study**

We identified symptoms that were or were not consistently associated with a positive SARS-CoV-2 test across various testing conditions by use of six datasets from three COVID-19 surveillance platforms in the USA, the UK, and Israel. These platforms are web-based and smartphone-based, as well as cross-sectional and longitudinal. The study period of 4 months covered varying COVID-19 prevalence during the fall of the first wave and, in some areas, rise of the second wave. Importantly, these collaborative analyses used large-scale surveillance data to track and highlight the value of individual symptoms, specifically anosmia, fever, and respiratory symptoms, to predict SARS-CoV-2 test positivity by region, platform, demographic factors, calendar time, timing of testing, illness duration, exposure and outcome ascertainment, and illness.

**Implications of all the available evidence**

Despite differences in syndromic surveillance methods, access to and timing of SARS-CoV-2 testing, and disease prevalence, anosmia or ageusia were consistently the strongest predictors of COVID-19 infection across all platforms over time. The odds of a positive COVID-19 test was nearly 17 times higher among individuals with anosmia or ageusia than those without these symptoms. Fever and respiratory symptoms (shortness of breath and cough) also ranked highly in their association with test positivity. This large, collaborative analysis showed that anosmia–ageusia, fever, shortness of breath, and cough are suitable empirical signals of ongoing COVID-19 transmission and could be particularly useful in regions where testing data are sparse or delayed. A prospective, iterative, surveillance data-based approach, using multiple datasets such as presented here, is likely to play an important role in other epidemiological contexts.

COVID-19 participatory surveillance platforms function in regions that have been variably affected by the pandemic, although no direct comparison of these data has been made to our knowledge. Testing policies,^15^ test access,^16^ and COVID-19-like illness (CLI) definitions have also varied substantially from country to country and over time. In many regions, testing was primarily targeted at individuals whose symptoms (or exposures) met strict criteria (eg, fever and respiratory symptoms)^17^ and then later, CLI symptoms were broadened to acknowledge the spectrum of COVID-19 presentations^18^ and to include other, sometimes highly specific, features (eg, anosmia).^19^

With all of these spatiotemporal changes in policies and access, as well as platform-specific study design features and inherent participation biases, we aimed to identify which symptoms were consistently associated with SARS-CoV-2 test positivity, and thus might represent the most clinically and epidemiologically relevant COVID-19 signals despite possible changes over time and across assessment types of their absolute effect estimates. To achieve this goal, we undertook a comparison of the association of putative CLI symptoms with self-reported SARS-CoV-2 testing results over time, by phase of illness, and in three countries across three citizen-science digital surveillance platforms.

## Methods

### Study design and population platforms

We used data from three participatory surveillance platforms in the USA, the UK, and Israel (two platforms per country), spanning a 4-month period of observation early in the pandemic (April 1 to July 31, 2020) to estimate odds ratios (ORs) for symptoms on self-reported SARS-CoV-2 test positivity among self-identified non-health-care workers (because health-care workers generally received different access to testing). Mapping of survey questions across platforms and survey language used is provided in the appendix (pp 10–15).

The Carnegie Mellon University (CMU; Pittsburgh, PA, USA) and University of Maryland (UMD; College Park, MD, USA) Facebook COVID-19 Symptom Survey (CMU/UMD) is one of the three participatory surveillance platforms providing the data used in this study, with respondents from the three study countries (the USA, the UK, and Israel). This survey was hosted by CMU's Delphi Research Center and provided web-based surveys to Facebook users,^20^ while UMD similarly coordinated surveys to Facebook users outside the USA.^21^ Surveys asked about geographical location, age, gender, working in a health-care setting, and the presence of symptoms in the preceding 24 h. Respondents who were symptomatic were additionally asked about SARS-CoV-2 test results. Test results from respondents outside the USA referred to tests in the preceding 14 days or, if ill, tests during the illness. Surveys are presumed to be from unique respondents based on the sampling strategy from Facebook US (50 US states and the District of Columbia), UK (Great Britain, excluding non-UK regions), and Israel. Survey-specific questions and logic are detailed in the appendix (pp 10–15). The survey was launched in the USA on April 6, 2020, and outside the USA on April 23, 2020. Anonymous surveys with non-missing self-reported age and sex and from respondents who did not work in a health-care setting were assessed for inclusion. Survey sampling strategies were used to increase representativeness of the source population for each nation by sampling from the Facebook active user base and ranking across census age, sex, and geographical region to develop survey weights. Data documentation for sampling methods have been published elsewhere.^20^ Primary analyses across all cohorts represent weighted parameters. Unweighted sensitivity analyses are detailed in the appendix (pp 1–2). This study was approved by the Boston Children's Hospital Institutional Review Board (P00023700).

The ZOE COVID Symptom Study App (ZOE) is another of the three participatory surveillance platforms providing the data used in this study, with respondents from the USA and the UK. The app was developed by ZOE Global (London, UK) with input from physicians and scientists from King's College London (London, UK), Massachusetts General Hospital (Boston, MA, USA), Lund University (Lund, Sweden), and Uppsala University (Uppsala, Sweden).^4^ The app was launched in the UK on March 24, 2020, and in the USA on March 29, 2020. At registration, users are asked for personal characteristics (age, gender, and whether they are a health-care worker). App users are asked through their mobile device to prospectively report their health status every day, indicating their symptoms if they have any. Additionally, they are asked to record their test results for COVID-19. Anonymised longitudinal, prospective collected trajectories of illness reports were available for app users for this study. Research studies on data collected through the app are approved by King's College London Ethics Committee REMAS ID 18210 (review reference LRS-19/20–18210), and all participants provided consent. Through a partnership between the UK Department for Health and Social Care, tests were made available to UK users of the app upon invitation from the app maintainers (ZOE) from April 26, 2020. By design, invited app users who logged being healthy twice in 9 days followed by an unhealthy report were invited to take a COVID-19 test. All test results were analysed in this study's main analysis. Multiple tests per user were censored within the symptom window following the test or once a test resulted positive.

The Corona Israel (Israel-Corona) study is the final participatory surveillance platform providing data for this study, with respondents from Israel. Israel-Corona data were collected through a voluntary online survey that included a 1-min, anonymous online questionnaire. The survey was first published on March 14, 2020.^5^ Survey responses were collected directly through the online platform. Responders were asked to report information on age, gender, geographical location, previous medical conditions, and whether they were a health-care worker, as well as symptoms occurring in the preceding 24 h for themselves and for each member of the family. Additionally, respondents were asked to report any SARS-CoV-2 testing and test results. This study was approved by the Weizmann Institute of Science Review Board. The Board waived informed consent as all identifying information was removed before the analysis.

### Study period and population criteria

Data from April 1 (or first testing data acquisition, if later) up to July 31, 2020, were aggregated into weeks, starting each Monday. The study population was restricted to respondents who self-reported a baseline age between 18 and 100 years (the CMU/UMD survey had decade age categories ranging from ≥18 years to ≥75 years), sex male or female, and non-health-care workers. For regression models, for the CMU/UMD survey age bins, the assigned age was the included decade (eg, 18–24 years as 20 years, 35–44 years as 40 years, ≥75 years as 80 years). Users with missing demographic data were excluded.

We reviewed publicly available data^15^, ^22^ regarding testing guidelines in each region during the study period. We specifically sought information regarding the shift in testing criteria from core CLI symptoms (ie, fever or respiratory symptoms) to a broader list of CLI symptoms. Open testing started on March 14, 2020, in the USA, whereas broader symptom-based testing occurred later in the UK (May 18, 2020) and Israel (June 1, 2020).^15^, ^19^ Additionally, these dates coincided with inclusion of anosmia–ageusia, except for the USA (April 5, 2020).

### Exposures (symptoms) and outcomes (COVID-19 test status)

We grouped 11 symptoms shared across at least two platforms into meta-symptoms (eg, myalgias or arthralgias inclusive of muscle pain and joint pain; appendix pp 10–15). Symptoms that were shared but had insufficient number of responses, and thus could not be compared (ie, abdominal pain, rash, or confusion), were excluded. Self-reported symptoms were considered present if logged within 14 days before the COVID-19 test (Israel-Corona, UK-ZOE, US-ZOE). For the USA, the UK, and Israel CMU/UMD cross-sectional survey, Facebook users were queried about symptoms present in the preceding 24 h, and symptomatic users were additionally asked about COVID-19 testing. In CMU/UMD surveys outside the USA, test status was queried for tests done during the course of the respective illness, or up to 14 days in the disease. To ensure privacy, Facebook users who responded to the cross-sectional survey did not contribute longitudinal data. We ran additional analyses in the US-ZOE and UK-ZOE surveys to assess the relevance of different symptoms when considering symptoms reported after a SARS-CoV-2 test, stratified by geographical region (USA *vs* UK), symptom onset-to-test duration (early [≤3 days] *vs* late [>3 days]), and periods of varying symptom criteria for testing access (narrow *vs* broad). We did sensitivity analyses of US-CMU/UMD data to assess the impact of illness duration on effect estimates.

The primary outcome was self-reported result of a SARS-CoV-2 test (ie, positive *vs* negative). Tests reported as pending or result unknown were excluded. Testing counts and positive test proportions were tabulated as the number of users (ZOE) or surveys (CMU/UMD and Israel-Corona) and the ratio of test positives to total tests reported with results. Multiple test results could be reported (Israel-Corona and ZOE); if multiple tests were done in a time window smaller than 14 days, only the first test was considered. Users were censored for a 14-day window or after a first positive test. US-CMU/UMD did not survey respondents regarding the timing of the test. CMU/UMD outside the USA specified test results within the duration of the respective illness, up to 14 days, or both, regardless of previous test results.

### Statistical analysis

We did logistic regression of each symptom (binary) on SARS-CoV-2 test status (binary) adjusted for age (continuous) and sex (binary) separately in each cohort. We calculated cross-correlations to assess the relationships between national and platform-specific measurements of tests and cases over time. We did meta-analyses assuming a random effects model (excluding diarrhoea with use of fixed effects due to fewer than five estimates to meta-analyse). We used robust rank aggregation to aggregate the rank lists of symptom–test positivity ORs. Cross-correlations of time series are reported. Analyses were done with R, version 3.6.3, glm for unweighted ORs, svyglm from the survey library for weighted ORs (CMU/UMD), rma from the metafor library for meta-analysis (random effects model specifying the restricted maximum-likelihood estimator via method=“REML”), aggregateRanks from the RobustRankAggreg library for rank (method=“RRA”) list aggregation, and python statsmodels, version 0.12.0 (Israel-Corona, ZOE).

### Role of the funding source

The funding sources had no role in study design, data collection, data analysis, data interpretation, or writing or the decision to submit the paper for publication.

## Results

Between April 1 and July 31, 2020, CMU/UMD registered 6 626 897 (USA), 272 767 (UK), and 98 540 (Israel) anonymous surveys with self-reported age and sex information from individuals who did not work in a health-care setting; ZOE counted 3 360 281 unique adult participants in the UK and 276 146 in the USA; and Israel-Corona registered 131 799 completed surveys from 29 993 unique users in Israel. Individuals participating in these surveillance platforms were more often women and tended to be younger and healthier than in the general population (table), a trend that is common in participants of technology-based, health-related surveys.^23^, ^24^, ^25^ Survey-weighted CMU/UMD cohort data were more representative of the source population (appendix p 16), but use of survey weights had little effect on results (appendix pp 1–2). Sensitivity analyses of demographic factors and adjustment effects for the UK-ZOE platform showed similar ranking of key symptoms (appendix p 8).

**Table:** Baseline characteristics of national platform users and survey respondents in relation to national government demographics

		**Israel**			**UK**			**USA**		
		Israel-Corona	Israel-CMU/UMD	Country	UK-ZOE	UK-CMU/UMD	Country	US-ZOE	US-CMU/UMD	Country
Number of adult individuals		29 993	98 540	6 129 363	3 360 281	272 767	52 261 668	276 146	6 626 897	255 271 738
Age of adult individuals, years		60·2 (15·9)	47·5 (17·1)	44·9 (18·5)	45·3 (15·6)	43·0 (15·6)	48·6 (18·6)	56·3 (16·3)	48·5 (16·3)	47·8 (18·3)
Gender										
	Men	15 257 (50·9%)	48 353 (49·1%)	2 993 325 (48·8%)	1 293 716 (38·5%)	100 536 (36·9%)	25 735 739 (49·2%)	93 910 (34·0%)	2 206 714 (33·3%)	124 267 346 (48·7%)
	Women	14 736 (49·1%)	50 187 (50·9%)	3 136 038 (51·2%)	2 066 565 (61·5%)	172 231 (63·1%)	26 525 929 (50·8%)	182 236 (66·0%)	4 420 183 (66·7%)	131 004 392 (51·3%)
Number of tests		16 531	1790	1 774 736	269 250	3410	9 415 384	24 286	199 192	62 092 416
Number of positive tests		40 (0·24%)	210 (11·7%)	70 379 (4·0%)	6037 (2·2%)	418 (12·3%)	302 301 (3·2%)	584 (2·4%)	28 355 (14·2%)	4 495 014 (7·2%)

Data are n, n (%), or mean (SD). Data on national demographics taken from the Israel Central Bureau of Statistics, the UK Office for National Statistics, and the US Census Bureau (2019 estimates). CMU/UMD data using survey weights is shown in the appendix (p 16). For cross-sectional CMU/UMD data, only tests with a positive or negative result are included, and the surveys queried users who were symptomatic. Pending or unknown test results were excluded. CMU/UMD=Carnegie Mellon University and University of Maryland Facebook COVID-19 Symptom Survey. Israel-Corona=Corona Israel study. ZOE=ZOE COVID Symptom Study app.

**Figure 1:**
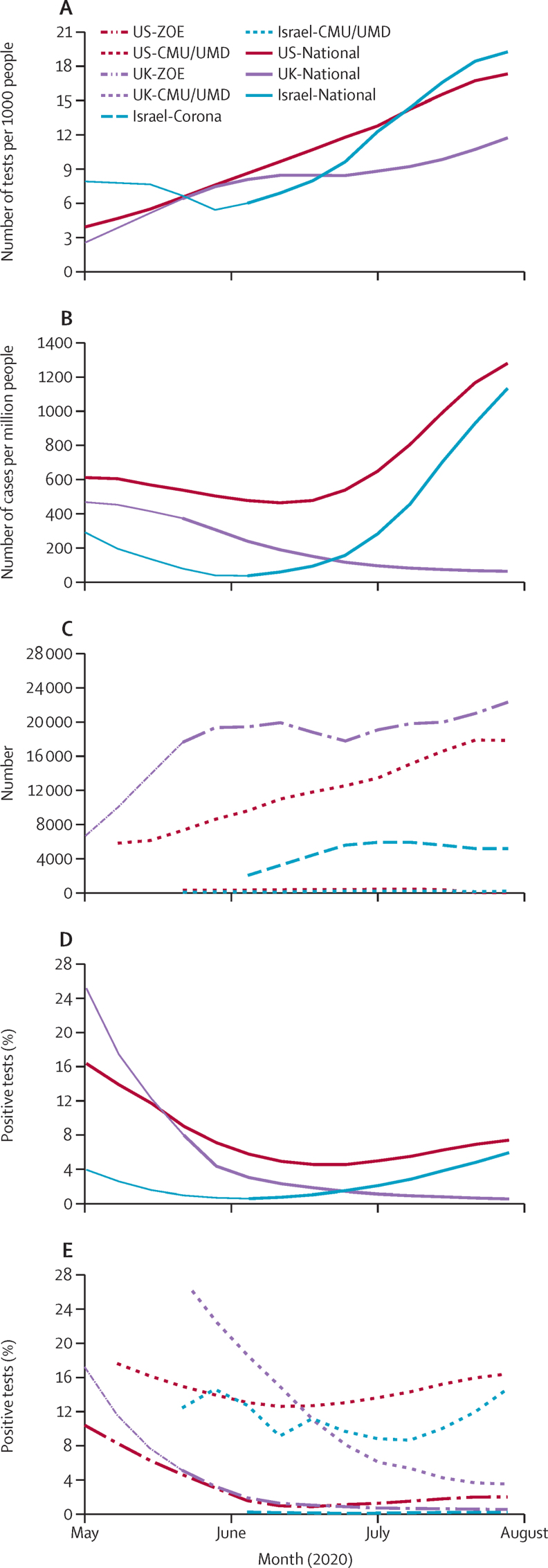
Weekly tests per person by country (A), cases per person by country (B), test results by platform (C), and proportion of positive tests by country (D) and platform (E) Data reported by platform during the study period in Israel (blue), the UK (purple), and the USA (red). National data shown as solid lines while surveillance platform data shown as dashed lines. The transition from thin to thick lines represents when testing policies were considered open. CMU/UMD=Carnegie Mellon University and University of Maryland Facebook COVID-19 Symptom Survey. Israel-Corona=Corona Israel study. ZOE=ZOE COVID Symptom Study app.

During the study period, SARS-CoV-2 testing capacity was scaled up (figure 1). Meanwhile, government-reported COVID-19 cases declined after April, 2020, (the first wave peak) due to a combination of interventions.^26^ COVID-19 cases recrudesced, first in Israel, and then in the USA (figure 1). In the UK, this second wave took place after the study period. Of the tests reported in the CMU/UMD cross-sectional surveys, 39 124 in US-CMU/UMD, 863 in UK-CMU/UMD, and 275 in Israel-CMU/UMD had results pending or unknown and were excluded from our analyses.

Trends in national testing data and proportion of positive tests were generally consistent with platform-specific tests reported (figure 1), with cross-correlations higher than 0·9 for testing (US-ZOE 0·97, UK-ZOE 0·96, US-CMU/UMD 0·99, UK-CMU/UMD 0·94, Israel-CMU/UMD 0·99) and higher than 0·8 for proportion of positive tests (US-ZOE 0·99, UK-ZOE >0·99, US-CMU/UMD 0·83, UK-CMU/UMD 0·94), except for testing (0·67) and proportion of positive tests (0·39) in Israel-Corona (the smallest study) and the proportion of positive tests in Israel-CMU/UMD (0·15). The median (range) proportion of positive tests across the six datasets was 7·05 (0·25–14·2). Although the CMU/UMD positivity proportion was higher than the national proportion (eg, US-CMU/UMD symptomatic test positivity is a subsample of all positive tests), the trend was representative (unweighted, incident–prevalent, and outlier sensitivity analyses are shown in the appendix, pp 1–4). Additionally, UK platform-led invitations for testing of individuals with any early symptom (appendix p 5) from early May, 2020, was followed by nationally mandated expansion of testing, accentuating the rise in tests reported in the app in May while slightly lowering the proportion of positive tests due to lower positivity in app users with mild symptoms than in the general population of app users. Many users invited for testing were at the early stages of their illness and had few symptoms (median two symptoms, IQR 1–4) at the time of invitation.

**Figure 2:**
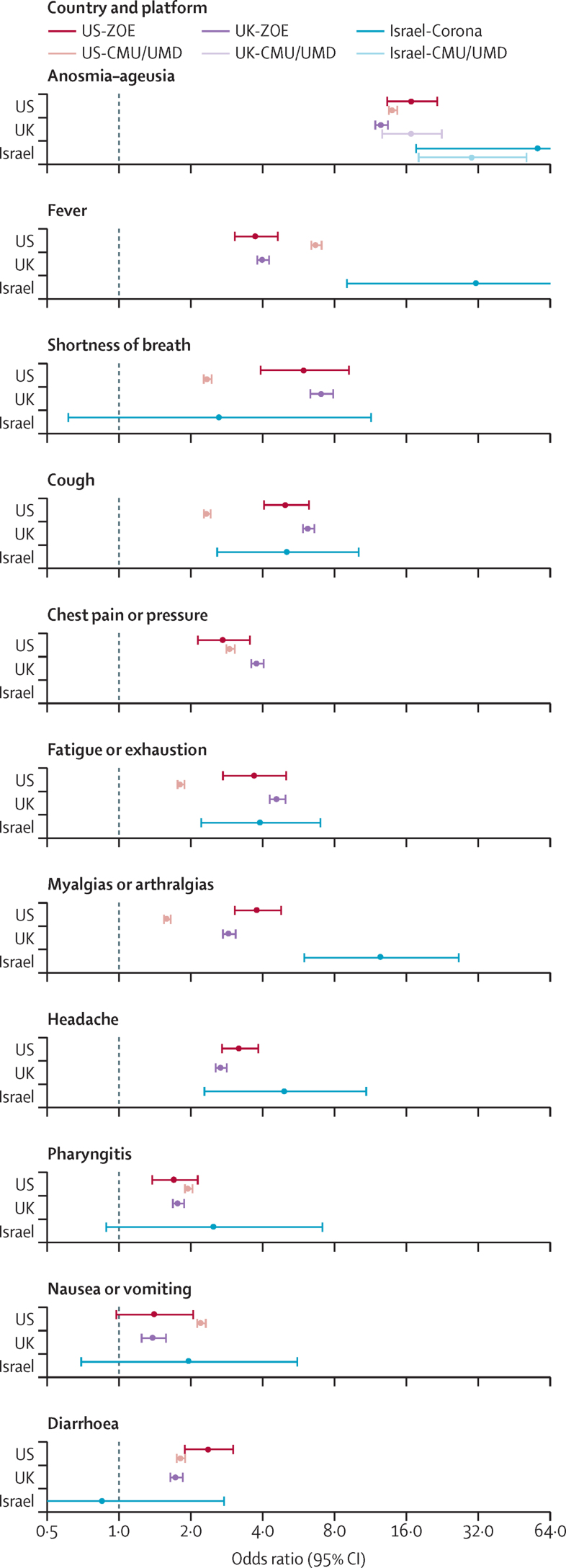
Comparison of odds ratios by country and platform for the outcome of test result (positive *vs* negative) for symptoms (facets) Sensitivity analyses, mapping, and survey language are shown in the appendix (pp 1–15). Odds ratio scale is log-linear to enable comparisons across a wide range of effect estimates. CMU/UMD=Carnegie Mellon University and University of Maryland Facebook COVID-19 Symptom Survey. Israel-Corona=Corona Israel study. ZOE=ZOE COVID Symptom Study app.

Symptom performance, as measured by the age-adjusted and sex-adjusted OR for the primary outcome of positive test versus negative test, showed consistently very elevated ORs for anosmia–ageusia (figure 2). Overall, anosmia–ageusia was an order of magnitude more common among individuals reporting positive test results (US-CMU/UMD 43%, UK-ZOE 29%, US-ZOE 19%, and Israel-Corona 14%) compared with those reporting negative test results (US-CMU/UMD 5%, UK-ZOE 2%, and Israel-Corona 0·2%), and became more prevalent in individuals testing positive as illness progressed (16% in UK-ZOE for invited users early in their illness compared with 44% for users with anosmia–ageusia up to 14 days after test result). The ORs were not constant over time and other variables, but the relative strength of anosmia–ageusia, fever, and respiratory symptoms was constant (appendix pp 1–9). We meta-analysed the six-country platform estimates for each symptom, as well as aggregated the ranks of each OR for the association of symptom with test positivity. Anosmia–ageusia had the strongest effect (random effects OR 16·96, 95% CI 13·13–21·92) and was the top ranked symptom (p <0·0001) by robust rank aggregation. Other core CLI components that were in the initial WHO CLI definition also ranked high, including fever (aggregated rank two), shortness of breath (rank three), and cough (rank four; figure 2). Broader testing criteria and a rise in cases in the USA (figure 2, appendix p 7) coincided with a rising OR for many symptoms (eg, Spearman's ρ 0·99 in US-CMU/UMD and 0·67 in US-ZOE for anosmia). The minimum OR for anosmia–ageusia (4·04, 95% CI 3·20–5·12) occurred during the lowest incidence of cases after the inclusion of this symptom in UK testing criteria on May 18, 2020.

Although CLI symptom signals were positive and similar, gastrointestinal symptoms were less consistently significantly associated. When restricting the analysis to individuals with few symptoms (oligosymptomatic here defined as five or fewer self-reported symptoms; appendix p 2), nausea and diarrhoea, along with myalgias or arthralgias and pharyngitis, were no longer predictive of test positivity. Similarly, gastrointestinal symptoms were equivocal in patients with shorter illness duration and during periods of low case incidence (in the UK).

**Figure 3:**
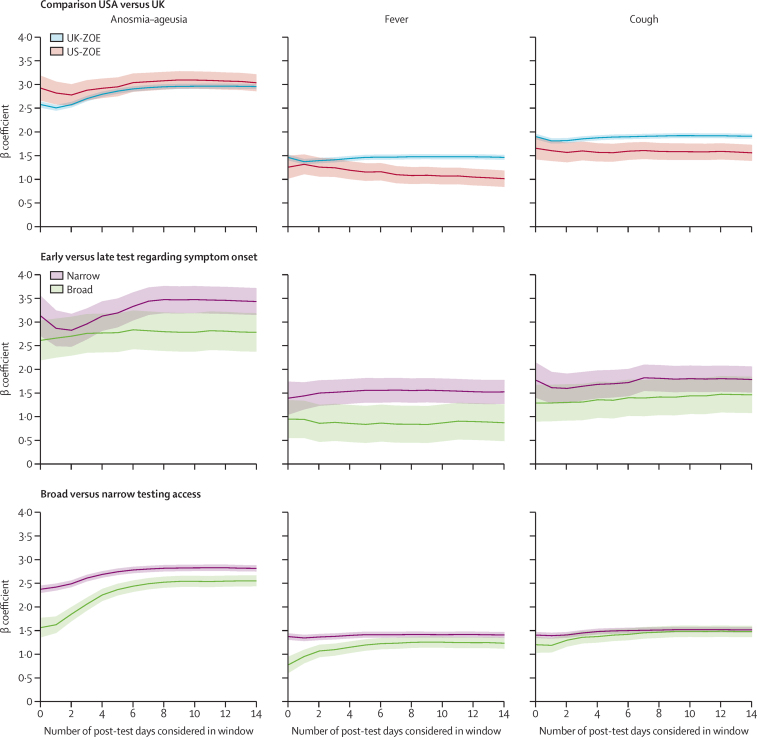
Longitudinal ZOE data stratified by country, time from symptom onset to test, and testing-qualifying symptom era Time from symptom onset to test was stratified as early (<3 days) versus late (≥3 days). Stratifications show the impact on effect estimates (y-axis) for the three canonical symptoms of anosmia–ageusia, fever, and cough. The x-axis gives the effect estimates when censoring symptoms 0–14 days after the reported COVID-19 test, which might include later-onset symptoms, as well as measurement bias resulting from the knowledge of the test result. ZOE=ZOE COVID Symptom Study app.

As expected, low incidence of positive cases generally coincided with wider CIs (figure 1, appendix p 7). The CMU/UMD Facebook active user base sampling scheme^20^ might have contributed to the more stable precision, although the timing of the tests relative to onset of specific symptoms cannot be ascertained. To evaluate whether symptom onset-to-test timing, illness duration, or recall bias (eg, US-CMU/UMD test and symptoms were surveyed simultaneously) affected symptom signals, we used the prospective, longitudinal follow-up of ZOE app users to investigate the change in OR signal when considering symptoms that are reported after a test, and early (up to 3 days) versus late (3 days or longer) in their illness when tested (3 days being the observed median time to get a test after symptom onset; figure 3). We also examined the timing of strictness of testing criteria (broad *vs* narrow). The OR for anosmia–ageusia, a later onset symptom, rose when up to 4 days of symptoms post-test were included, although this rise was smaller for people tested later in their illness and greater when the UK broadened the symptom criteria for testing. We compare this with CMU/UMD stratified by illness duration (appendix pp 3–4), which showed the peak OR for anosmia–ageusia at 14 days from symptom start.

## Discussion

In this study, we showed convincing evidence that self-reported anosmia–ageusia is the most robustly associated symptom with SARS-CoV-2 test positivity, regardless of the surveillance platform used or population, testing guidelines or capacity, illness duration or complexity, or timing of testing. This supports results from previous studies and the initial (March 24 to April 21, 2020) US and UK ZOE symptom score analysis, which have focused on single platforms, countries, or time periods.^14^, ^27^, ^28^, ^29^ Anosmia–ageusia was overall more common among individuals reporting positive test results than among those reporting negative test results, and became more prevalent in those testing positive as illness progressed. This finding supports test access and self-isolation mandates at the onset of anosmia–ageusia.^19^, ^22^, ^30^

Core CLI components of fever, cough, and shortness of breath similarly performed well under a wide range of scenarios evaluated. Importantly, although symptom associations varied across platforms, the top performing symptoms were consistently anosmia–ageusia, fever, and cough and shortness of breath. Other symptoms were inconsistent predictors, or most relevant under specific circumstances. These findings highlight key COVID-19 symptoms as signals for multiregional syndromic surveillance under various surveillance platform designs. Testing is a cornerstone of the pandemic response that has presented substantial challenges globally.^10^, ^16^ Having a set of generalisable CLI signals is particularly important for global public health efforts where government data on COVID-19 incidence are sparse or delayed, or where region-specific benchmarking or fine-tuning of CLI prediction models might not be possible. Our findings support the use of anosmia–ageusia, fever, cough, and shortness of breath as reasonable, empirical signals for surveillance in these settings.

These findings show the power of using a digital interface to collect epidemiological data on a multinational scale, tailored to public health needs (eg, longitudinal disease trajectory and consistent or representative population sampling) over space and time, in the response to a novel pathogen. Although privacy limits the validation of anonymous self-reports against health records, the near-real-time, survey-based outcomes closely mirror national trends and are thus useful for so-called nowcasting and forecasting.^31^, ^32^ As is the case in other fields such as genomics, this new multiplatform collaboration to compare and combine effect estimates enhances our understanding of COVID-19 epidemiology, while also validating features of individual studies. Although no surveillance platform is immune from biases, together these platforms highlight consistent COVID-19 features that are apparent despite the cross-sectional, opt-in nature, and other platform-specific features. Additionally, the differences in the effect estimates also reveal important aspects of COVID-19 surveillance to consider as the pandemic evolves. For example, active invitation to test from a platform has the potential to capture individuals with symptomatic infection earlier than government-invited testing, even though symptoms of brief duration at the time of testing might be less predictive of test positivity. The importance of pharyngitis and gastrointestinal symptoms, for example, might be for individuals with multiple symptoms at presentation. We hypothesise that these findings might be due to clustering of symptoms or the phase of illness when testing was completed. The CMU/UMD Facebook active user base sampling scheme^20^ might have contributed to the more stable precision, although the timing of the tests relative to onset of specific symptoms cannot be ascertained. Future directions for this type of collaboration could include discriminating COVID-19 from seasonal respiratory pathogens such as influenza,^27^, ^33^ although few datasets^1^, ^2^ exist from which to define discriminating symptoms a priori. A prospective, iterative, surveillance data-based approach, using multiple datasets such as that done here, is likely to play an important role.

Our study has some limitations. These findings should be interpreted with the caveat that, by its nature, real-time participatory syndromic surveillance inherently has potential biases related to, for example, generalisability and selection bias (eg, whether participants are representative of the source population, participation is differential regarding exposure or outcome, or the platforms have covariates for crucial effect modifiers), and measurement bias (eg, survey question misunderstanding, differential missing data or error in self-reporting due to incentive to record being healthy when being monitored, survey misuse, or one-time surveys without longitudinal follow-up of future outcomes). We compared each platform with national demographics and outcomes, as well as survey-weighted outcomes (for CMU/UMD). For both UK-ZOE and US-CMU/UMD platforms, respondents were younger and more often women than the general population, which is similar to published online survey participation demographics and echoes research showing possible biases related to use of mobile health devices and solutions in the context of symptom reporting in the COVID-19 era.^23^, ^24^, ^25^ Sensitivity analyses within demographic subgroups showed differences in the absolute but not relative associations of canonical symptoms.

For this interplatform international comparison of symptom-based COVID-19 prediction, we had to map survey questions (eg, subjective fever *vs* temperature threshold) and account for study design variation (eg, US-CMU/UMD queried symptoms over the 24 h before any test result, whereas Israel-Corona included symptoms logged 14 days before the test report). However, we should note that due to the necessary broad encapsulation of symptoms enumerated in each platform, the reporting of all symptoms, including anosmia–ageusia, might reflect subjective interpretations rather than clinical features and might not encompass related symptoms that might be even more highly associated with COVID-19, such as dysgeusia.

To address measurement bias, we compared symptoms test windows and phase of illness. Similarly, while these design choices affected the magnitude of effect estimates, the overall trends and the strength of anosmia–ageusia and core CLI symptom–test associations remained evident. Sensitivity analyses showed our findings to be robust to relaxing assumptions such as illness duration, symptom-to-test window, symptom report pattern, platform-suggested testing, and the use of survey weights. The possibility of one individual being tested multiple times over the course of the disease was beyond the scope of this study and not feasible with one-time surveys. Our study cannot assess clinical evaluation of specific symptoms (eg, fever measured by a thermometer or true anosmia assessed by a smell test) in relation to the users' subjective perception. However, many screening tools in use rely on a person's self-report of symptoms.

Despite these limitations, the strength of this study lies in the combination of data from very different digital platforms that vary in terms of their participants' location (Israel, the UK, and the USA), assessment design, and their observation over time (April to July, 2020). All six datasets combined are very large in size (over 10 million respondents), with high numbers of tests done (over half a million) and the capacity to provide automated, aggregate outcomes in near-real time. We were able to show within and between platform and country the associations of CLI symptoms with COVID-19 test positivity. Lastly, we present here evidence for the use of CLI signals for surveillance of anosmia–ageusia, fever, and respiratory symptoms for surveillance in regions for which real-time COVID-19 case data are inadequate.

To our knowledge, this is the first comparison of COVID-19-associated symptoms across multiple countries and surveillance cross-platforms of this scale. We established the strength of fever and respiratory symptoms as good CLI signals, with some variation regarding which respiratory symptom was most associated with COVID-19. Importantly, we showed the generalisability of the unique symptom of anosmia–ageusia as the single strongest predictor of all CLI symptoms considered.

**This online publication has been corrected. The corrected version first appeared at thelancet.com/digital-health on August 23, 2021**

## Data sharing

Tables of de-identified, aggregated data for the Israel-Corona platform are available at https://github.com/hrossman/Covid19-Survey. Data from the ZOE platform used in this study are available to researchers through the UK Health Data Research at https://web.www.healthdatagateway.org/dataset/fddcb382-3051-4394-8436-b92295f14259. Requests for access to the CMU/UMD Facebook COVID-19 Symptom Survey can be done at https://dataforgood.fb.com/docs/covid-19-symptom-survey-request-for-data-access/.

## Declaration of interests

ZOE Global codeveloped the app pro bono for non-commercial purposes. JW, JCP, and SG work for ZOE Global, and TDS is a consultant for ZOE Global. LHN, DAD, and ATC previously participated as investigators on a diet study unrelated to this work, which was supported by ZOE Global. ATC reports personal fees from Pfizer, Bayer Pharma, and Boehringer Ingelheim, outside the submitted work. All other authors declare no competing interests.
