
JOURNAL INFORMATION
==============================
NLM Title Abbreviation: Lancet Digit Health
Journal ID: Lancet Digit Health
NLM Journal ID: 101751302

The Lancet. Digital Health

EISSN: 2589-7500

ARTICLE INFORMATION
==============================
PMCID: PMC8382286
PMID: 34446264
DOI: 10.1016/S2589-7500(21)00181-3
Article ID: S2589-7500(21)00181-3
Article version: 2
Subjects: Corrections

Correction to Lancet Digit Health 2021; 3: e577–86

Publication date: 2021 Sep
Electronic publication date: 2021 Aug 23
Volume: 3
Issue: 9
First page: e542
Last page: e542
Copyright: © 2021 The Author(s). Published by Elsevier Ltd.
Copyright year: 2021
Copyright holder: Elsevier Ltd
License: This is an open access article under the CC BY license (http://creativecommons.org/licenses/by/4.0/).
License URL: https://creativecommons.org/licenses/by/4.0/

Erratum for: Anosmia, ageusia, and other COVID-19-like symptoms in association with a positive SARS-CoV-2 test, across six national digital surveillance platforms: an observational study 3 9 22 7 2021 e577 e586 The Lancet. Digital Health 10.1016/S2589-7500(21)00115-1 PMC8297994 34305035

==============================
Sudre CH, Keshet A, Graham MS, et al. Anosmia, ageusia, and other COVID-19-like symptoms in association with positive SARS-CoV-2 test, across six national digital surveillance platforms: an observational study. Lancet Digit Health 2021; 3: e577–86—In this Article, the spelling of author Erika Molteni's name was incorrect. This correction has been made as of Aug 23, 2021.

