# Supplementary material for: Anosmia, ageusia, and other COVID-19-like symptoms in association with a positive SARS-CoV-2 test, across six national digital surveillance platforms: an observational study
Source: Lancet Digit Health. 2021 Jul 22;3(9):e577–86. doi: 10.1016/S2589-7500(21)00115-1 (PMC8297994; doi:10.1016/S2589-7500(21)00115-1)
Supplement: Supplementary appendix [file mmc1.pdf]

THE LANCET
Digital Health

Supplementary appendix

This appendix formed part of the original submission and has been peer reviewed. We post it as supplied by the authors.

Supplement to: Sudre CH, Keshet A, Graham MS, et al. Anosmia, ageusia, and other COVID-19-like symptoms in association with a positive SARS-CoV-2 test, across six national digital surveillance platforms: an observational study. *Lancet Digit Health* 2021; published online July 22. https://doi.org/10.1016/S2589-7500(21)00115-1.

Supplementary material - Web material

Supplementary figure 1: CMU/UMD platform weights and filtering sensitivity analysis

a) CMU/UMD platform odds ratios (95% confidence intervals) sensitivity analyses with and without survey weights for US-CMU/UMD, UK-CMU/UMD and Israel-CMU/UMD for the entire study period. Additional sensitivity analyses with US-CMU/UMD showing (b) number of reported tests and positive tests and (c) moving 5-week Odds Ratios effect estimates alongside sensitivity analyses with strict filtering (including surveys with no more than 5, 10 and 13 of 14 symptoms reported during the prior 24 hours), or with survey weights (all other OR are unweighted). For survey weight analyses, Facebook Active User Base “raked” to the national census population[34].

(a) Sensitivity analysis of raw vs survey-weighted OR by country

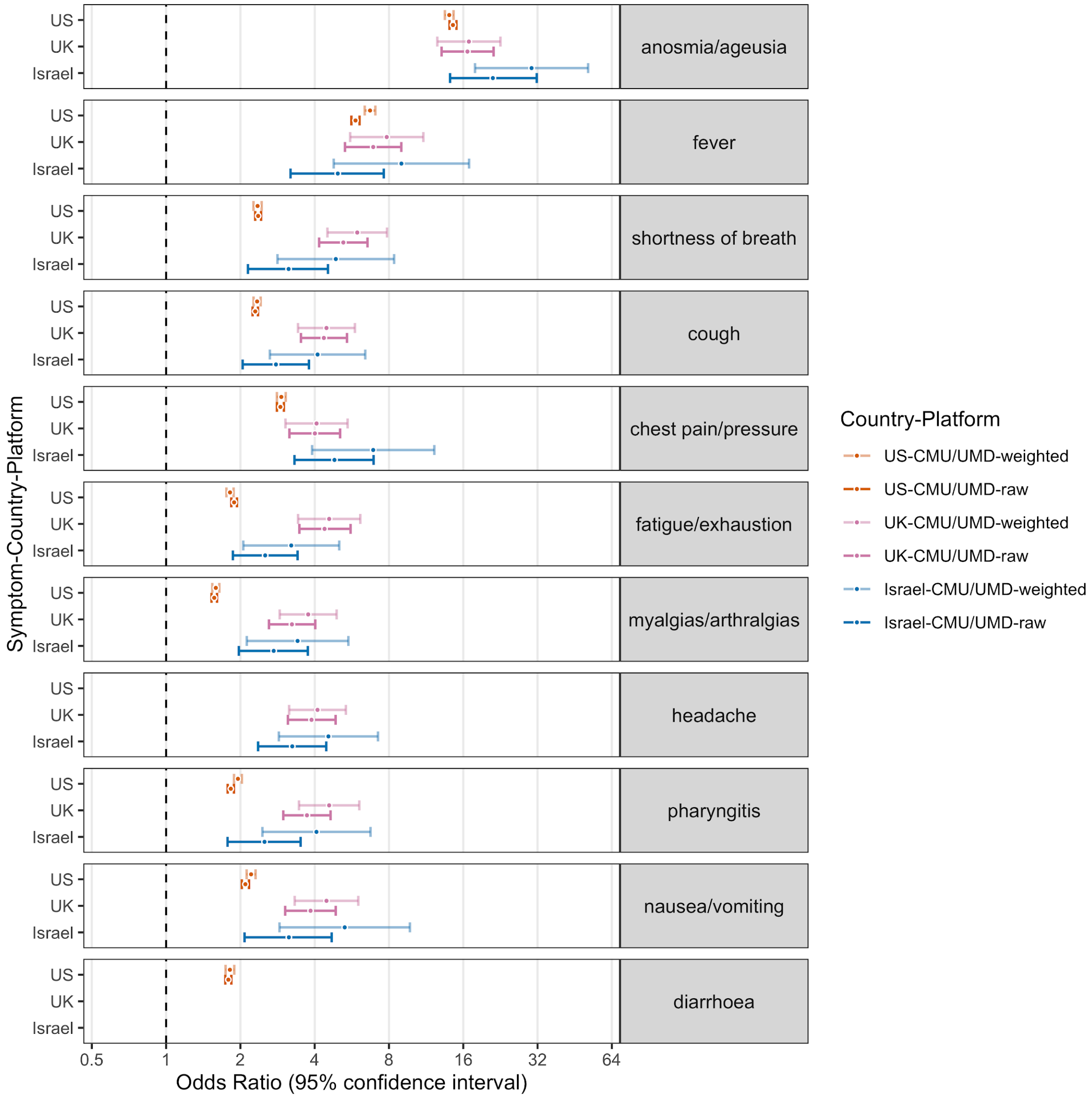

(b) Sensitivity analysis of test positivity for survey-weighted vs raw with symptom-count filters over time.

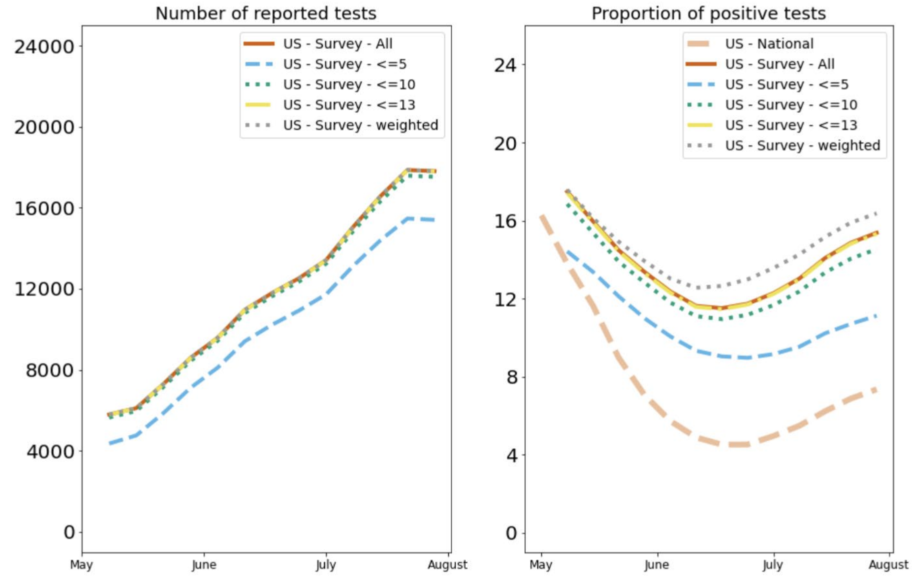

(c) Sensitivity analysis of OR for survey-weighted vs raw with symptom-count filters over time.

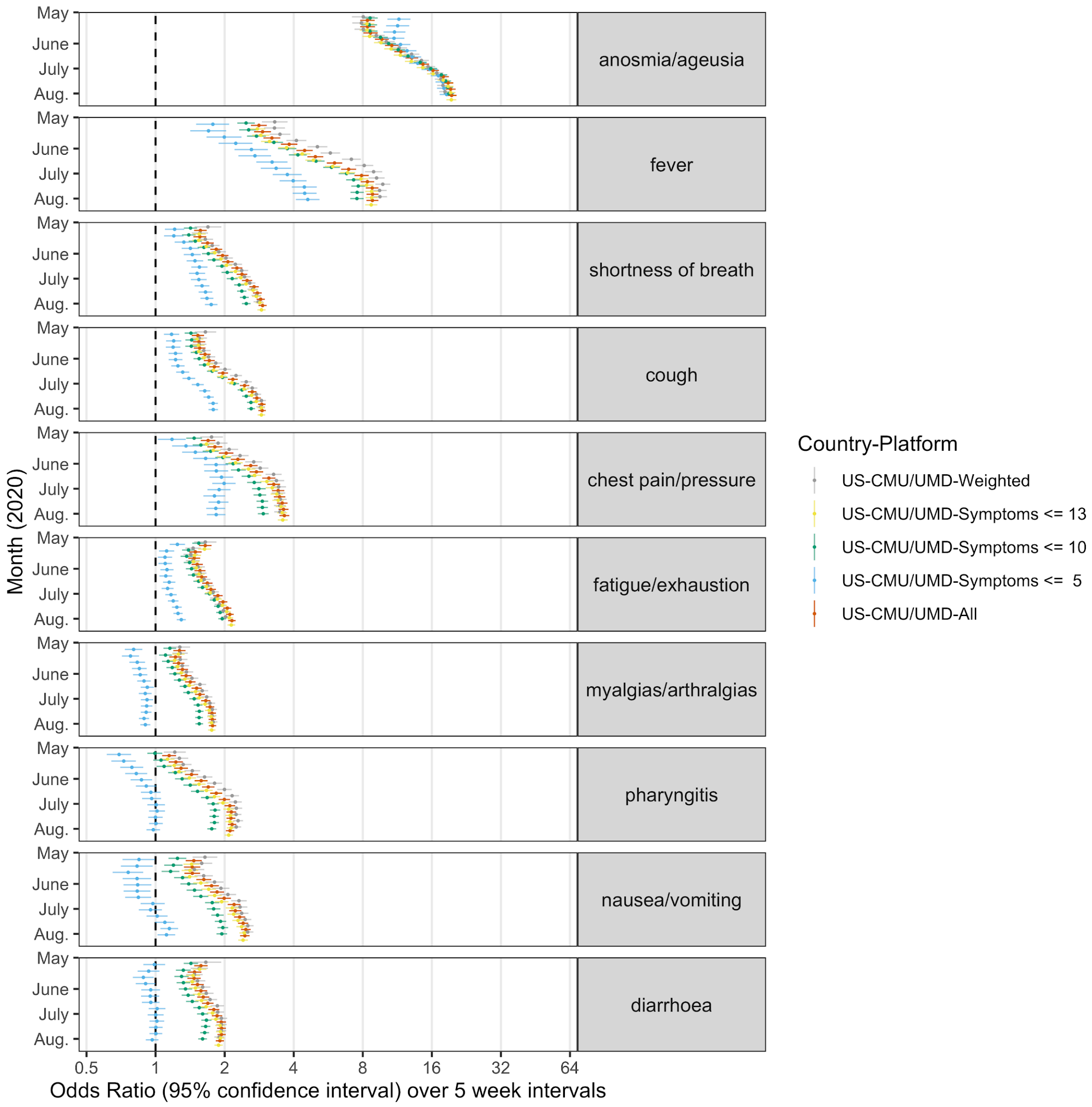

May
June
July
Aug.
anosmia/ageusia
fever
shortness of breath
cough
chest pain/pressure
fatigue/exhaustion
myalgias/arthralgias
pharyngitis
nausea/vomiting
diarrhoea
Month (2020)
0.5
1
2
4
8
16
32
64
Odds Ratio (95% confidence interval) over 5 week intervals
Country-Platform
US-CMU/UMD-Weighted
US-CMU/UMD-Symptoms <= 13
US-CMU/UMD-Symptoms <= 10
US-CMU/UMD-Symptoms <= 5
US-CMU/UMD-All

Supplementary figure 2: CMU/UMD illness duration sensitivity analysis

Sensitivity analyses of CMU/UMD demonstrating short (14 days) versus long (21 days) symptom-to-test window or varying illness duration during the study period. In CMU/UMD, a cross-sectional survey, illness duration of 3, 7, 14 and 21. The timing of the test relative to symptoms is unknown in the US-CMU/UMD while the test result reported was during the period of illness in the ex-US-CMU/UMD data (capped at 14 days). Most symptoms have higher associations with test positivity.In addition, (b) we show the trajectory of test positive less than 14 and 21 days in US-CMU/UMD in 5 week intervals is more similar to the national pattern, reflecting more incident than prevalent illness with test results. Sensitivity OR are unweighted.

(a) Sensitivity analysis of OR for illness duration

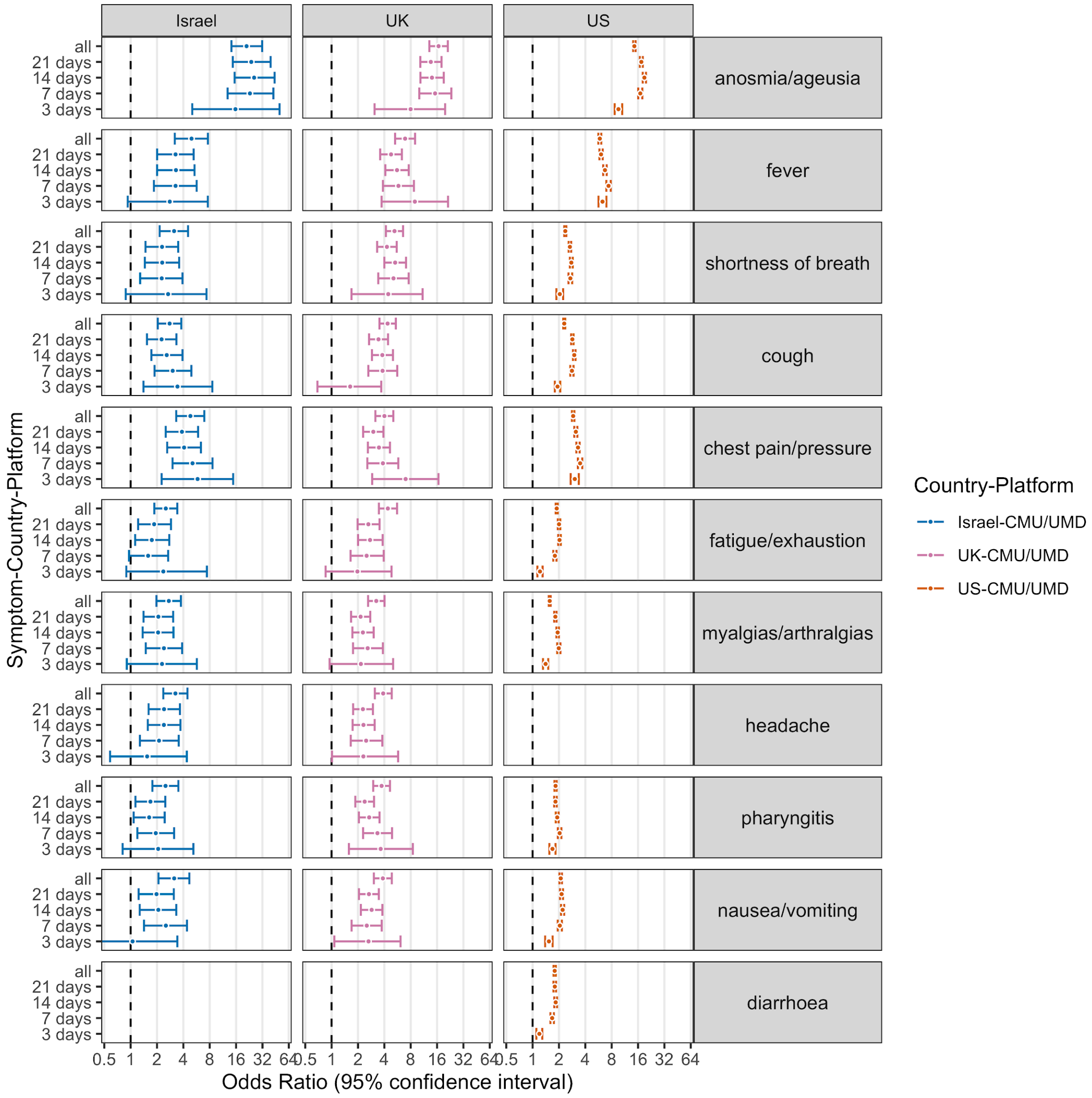

(b) Sensitivity analysis of test positivity for illness duration

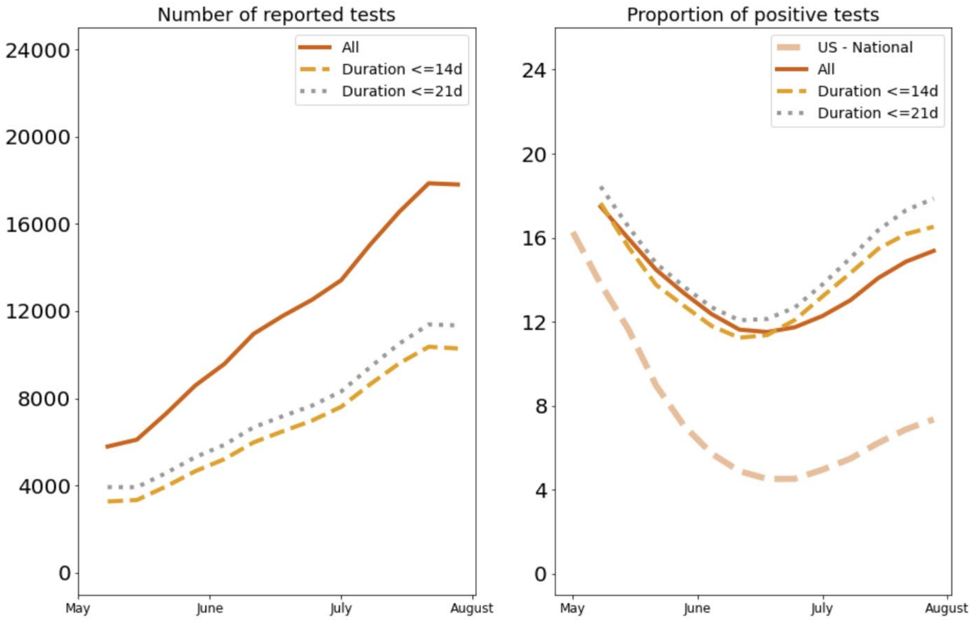
Number of reported tests
All
Duration <=14d
Duration <=21d
24000
20000
16000
12000
8000
4000
0
May
June
July
August
Proportion of positive tests
US - National
All
Duration <=14d
Duration <=21d
24
20
16
12
8
4
0
May
June
July
August

Supplementary figure 3: UK-Zoe invited testing sensitivity analysis

In the UK-Zoe study, beginning 29 April 2020, oligosymptomatic individuals were invited to participate in platform-sponsored SARS-CoV-2 PCR testing, regardless of whether they met testing guidelines in the region. In the UK-Zoe platform primary analysis, invited-test respondents and not invited-test were combined. Here we show test positivity and OR stratified by whether respondents were or were not invited to test. For those with repeated tests, negative test results following a positive test were censored. Invited data are plotted starting from the first 5-week bin, while the full data are plotted from study inception (which includes increasing numbers of invited tests). Panel (a) number of reported tests and positive tests for each 5-week bin, and panel (b) odds ratios for the study period for UK-Zoe participants invited and not-invited to test with few symptoms, alongside the combined analysis..

(a) Sensitivity analysis of test positivity for invited and non-invited test UK-Zoe participants

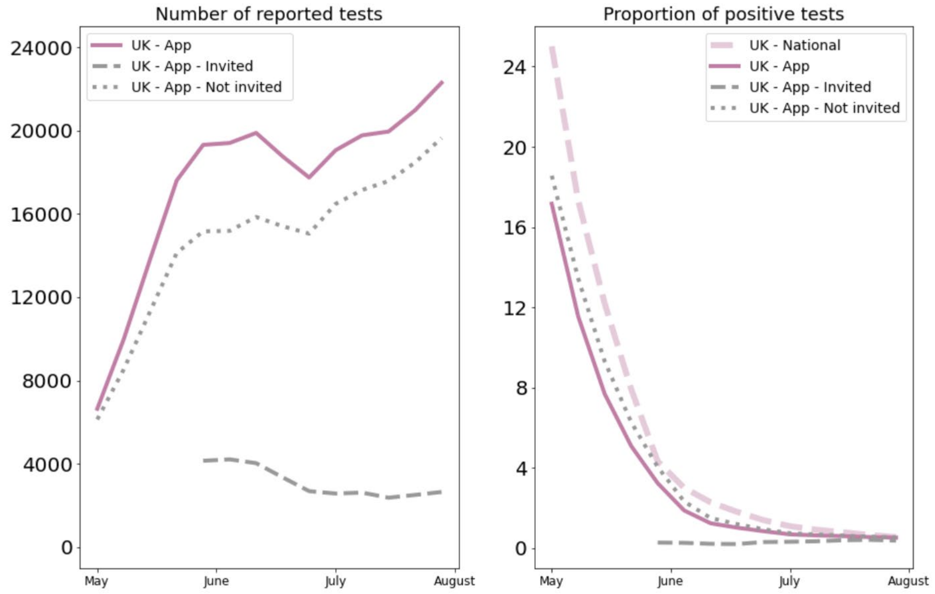

(b) Sensitivity analysis of OR for invited and not-invited test UK-Zoe test participants

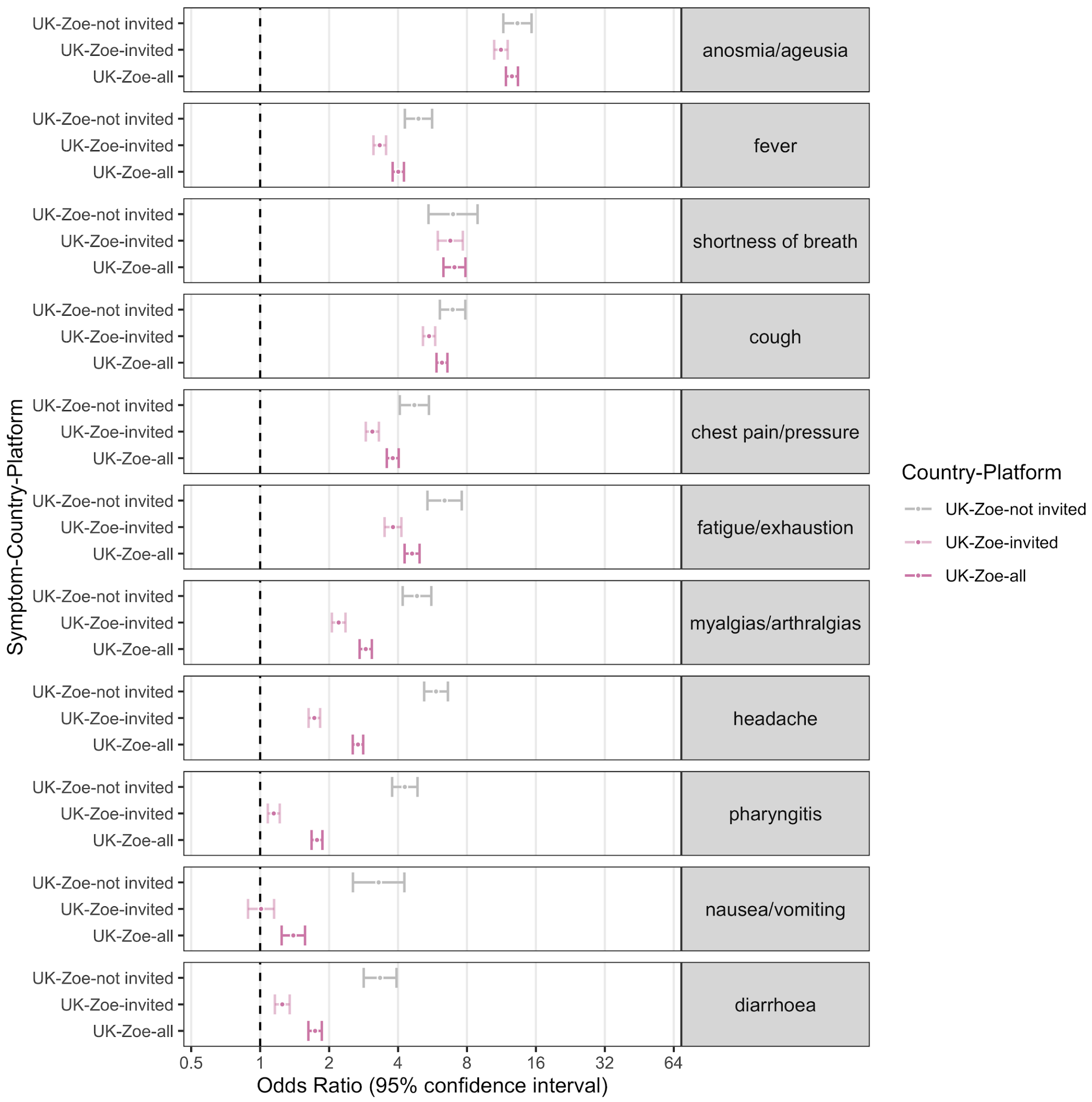
UK-Zoe-not invited
UK-Zoe-invited
UK-Zoe-all
anosmia/ageusia
fever
shortness of breath
cough
chest pain/pressure
fatigue/exhaustion
myalgias/arthralgias
headache
pharyngitis
nausea/vomiting
diarrhoea
Symptom-Country-Platform
0.5
1
2
4
8
16
32
64
Odds Ratio (95% confidence interval)
Country-Platform
UK-Zoe-not invited
UK-Zoe-invited
UK-Zoe-all

Supplementary Figure 4: Temporal trends in symptom-test positivity association

Country-platform data aggregated over 9-week windows to show temporal trends with expansion of testing, broadening of testing criteria and change in prevalence during the study period. Sensitivity OR for CMU/UMD are unweighted.

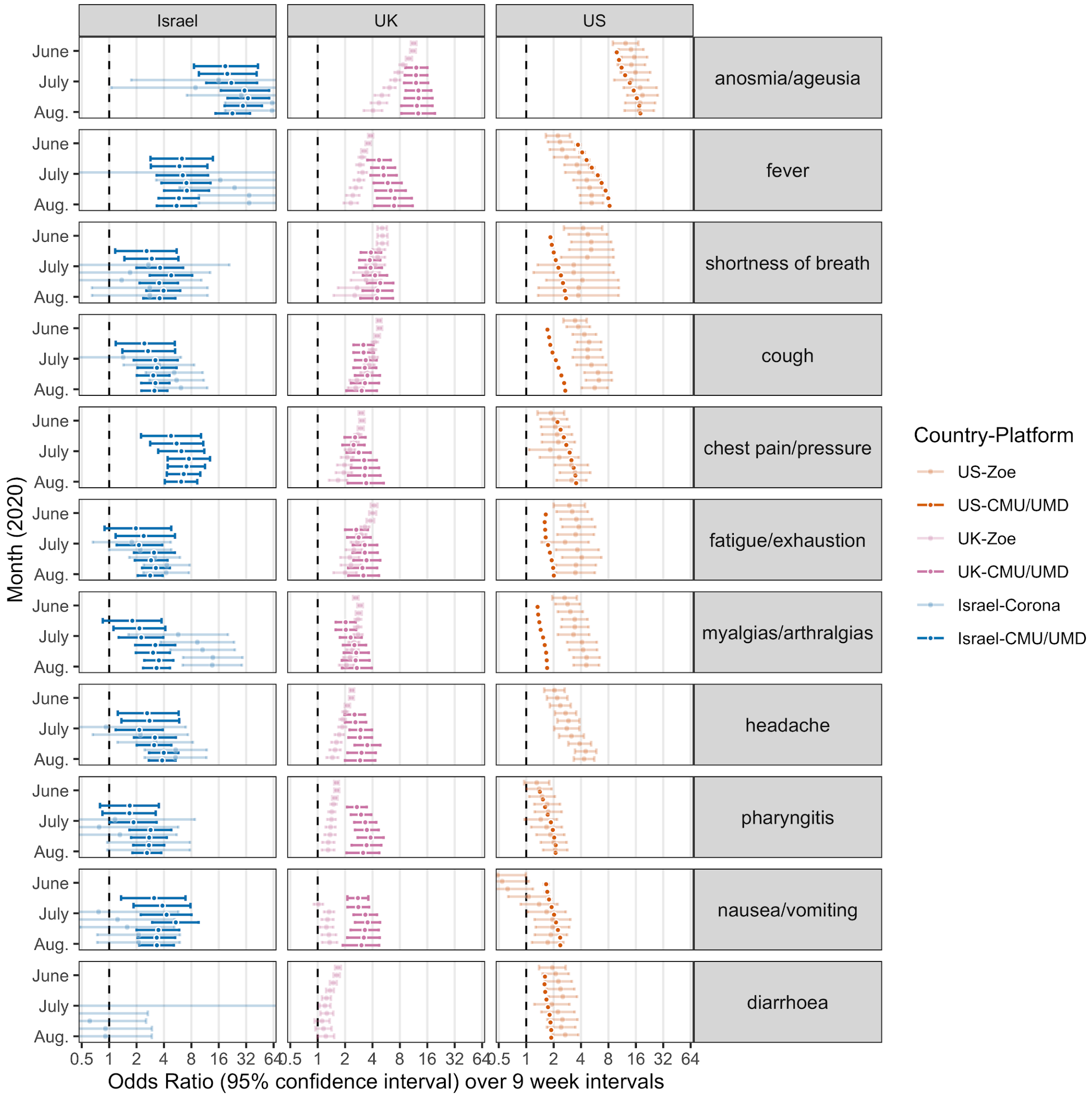

Supplementary figure 5: Sensitivity analysis of opt-in platform demographics.

UK-Zoe effect estimates by region for three canonical COVID-19 (a) and age-sex strata over time using 9-week intervals (b). Although there are differences in the width of confidence intervals and magnitude of the point estimates, the relative importance of these three symptoms are consistent, i.e. anosmia/ageusia the most strongly associated with test positivity.

(a) Sensitivity analysis of effect estimates for anosmia/ageusia, fever and cough by geographic region (top) and age-sex strata (bottom)

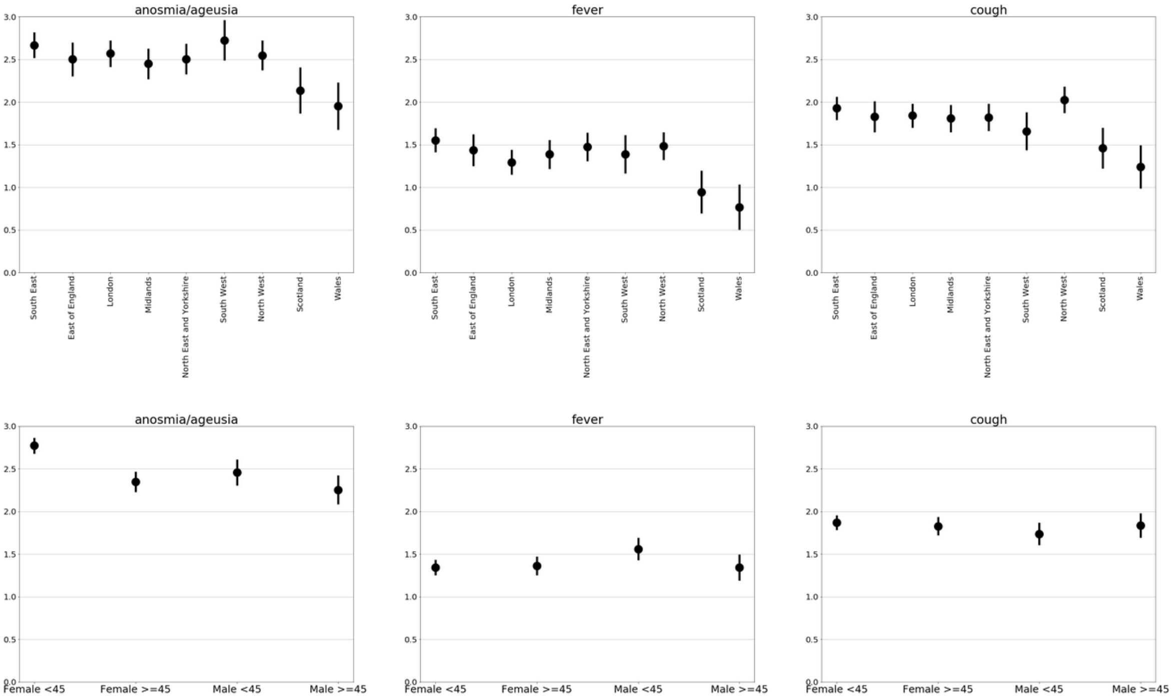

(b) Sensitivity analysis of effect estimates over time with and without adjusting for age and sex as covariates

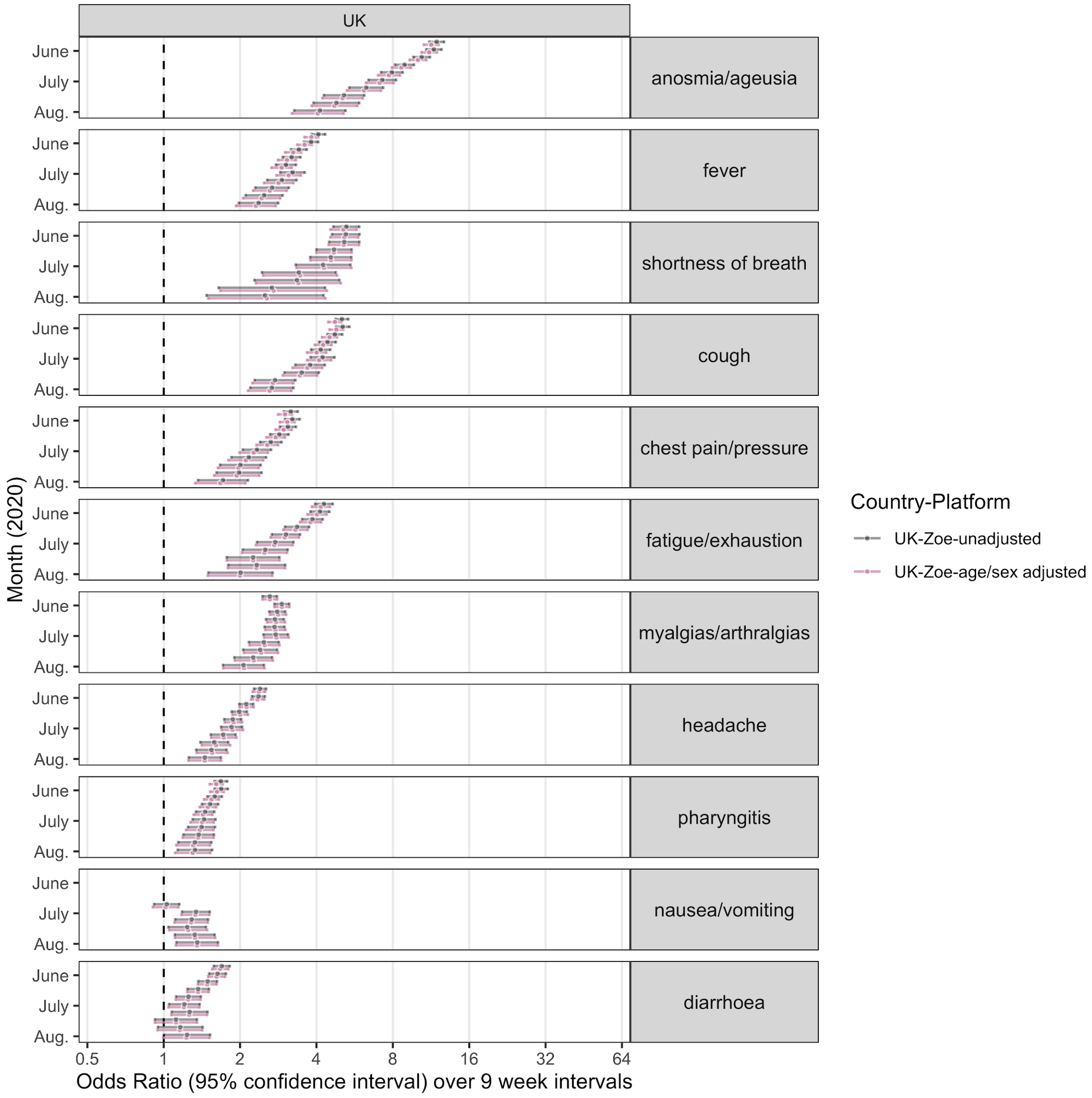
UK
June
July
Aug.
anosmia/ageusia
fever
shortness of breath
cough
chest pain/pressure
fatigue/exhaustion
myalgias/arthralgias
headache
pharyngitis
nausea/vomiting
diarrhoea
Month (2020)
0.5
1
2
4
8
16
32
64
Odds Ratio (95% confidence interval) over 9 week intervals
Country-Platform
UK-Zoe-unadjusted
UK-Zoe-age/sex adjusted

Supplementary table 1: Mapping of survey questions across platforms

Mapping of questions and symptoms across datasets. Eleven symptoms were shared across at least two of three platforms, after having excluded symptoms that were shared but had limited responses, and thus could not be compared (i.e. abdominal pain, rash, confusion). CMU/UMD english language survey translated into multiple languages for ex-US users.

Label	Survey	Questions asked	Note / Date of change
Unhealthy report	US-CMU/UMD	In the past 24 hours, have you personally experienced any of the following symptoms? (Select all that apply.)	
	Ex-US-CMU/UMD	In the last 24 hours, have you had any of the following?	
Anosmia/ ageusia	US-CMU/UMD	Loss of smell or taste	
	Ex-US-CMU/UMD	Loss of smell or taste	
	CSS UK	Do you have a loss of smell/taste	
	Israel Corona	Are you experiencing loss of taste and/or smell sensation	
Fever	US-CMU/UMD	Fever	
	Ex-US-CMU/UMD	Fever	
	CSS UK	Do you have a fever or feel too hot	
	Israel Corona	What is your current body temperature?<br>☐ I did not measure my temperature in the last 24 hours<br>☐ I measured my temperature and it was _____	
Chest pain	US-CMU/UMD	Persistent pain or pressure in your chest	
	Ex-US-CMU/UMD	Chest pain	
	CSS UK	Are you feeling an unusual chest pain or tightness in your chest	
	Israel Corona	NA	NA
Short of	US-CMU/UMD	Shortness of breath	

breath	Ex-US-CMU/UMD	Difficulty breathing	
	CSS UK	Are you experiencing unusual shortness of breath	
	Israel Corona	Are you experiencing shortness of breath	
Cough	US-CMU/UMD	Cough	
	Ex-US-CMU/UMD	Cough	
	CSS UK	Do you have a persistent cough (coughing a lot for more than an hour or 3 or more coughing episodes in 24 hours)	
	Israel Corona	Are you experiencing<br>☐ Dry cough (no sputum)<br>☐ Wet cough (with sputum)	
Nausea / Vomit	US-CMU/UMD	Nausea or vomiting	
	Ex-US-CMU/UMD	Nausea	
	CSS UK	Have you felt nauseous or experienced vomiting	29 April 2020
	Israel Corona	Are you experiencing nausea and /or vomiting	
Diarrhoea	US-CMU/UMD	Diarrhea	
	Ex-US-CMU/UMD	NA	
	CSS UK	Are you experiencing diarrhoea	
	Israel Corona	Are you experiencing diarrhea	
Fatigue	US-CMU/UMD	Tiredness or exhaustion	
	Ex-US-CMU/UMD	Fatigue	
	CSS UK	Are you experiencing unusual fatigue	
	Israel Corona	Are you experiencing fatigue	

Myalgia/arthralgia	US-CMU/UMD	Muscle or joint aches	
	Ex-US-CMU/UMD	Aches or muscle pain	
	CSS UK	Do you have unusual strong muscle pains	
	Israel Corona	Are you experiencing muscle pain	
Headache	US-CMU/UMD	NA	
	Ex-US-CMU/UMD	Headache	
	CSS UK	Do you have a headache	
	Israel Corona	Are you experiencing headache	
Pharyngitis	US-CMU/UMD	Sore throat	
	Ex-US-CMU/UMD	Sore throat	
	CSS UK	Do you have a sore throat	
	Israel Corona	Are you experiencing sore throat	
Test question	US-CMU/UMD	[Asked to respondents who report at least 1 of 14 symptoms]<br>Have you been tested for COVID-19 (coronavirus) for your current illness?<br>☐ Yes, I was tested, and received a positive diagnosis for COVID-19 (1)<br>☐ Yes, I was tested, but it was negative for COVID-19 (2)<br>☐ Yes, I was tested, but have not received the result (3)<br>☐ No, I tried to get tested but could not get a test (4)<br>☐ No, I have not tried to get tested (5)	
	Ex-US-CMU/UMD	Have you ever been tested for coronavirus (COVID-19)? 1=Yes, 2=No<br>[If Yes, respondents are asked]<br>Have you been tested for coronavirus (COVID-19) in the last ${e://Field/sympdays} days?	

		[where sympdays field is from the question is asked to respondents who report at least 1 of 12, (through 7 May 2020) or of 13 symptoms] For how many days have you had at least one of these symptoms? [capped at 14 days]<br><br>[If Yes, respondents are asked]<br>Did your most recent test find that you had coronavirus (COVID-19)?<br>1=Yes<br>2=No<br>3=I don't know	
	CSS UK	Initial test question:<br>Have you had a Covid Test - If yes:<br>Did you test positive<br>Date inferred based on the date of result<br><br>Update in question:<br>Have you had a new Covid Test<br>If yes -<br>How was the test performed (description of test to infer whether antibody or swab test)<br>What was the result<br>When was your test	
	Israel Corona	Online survey:<br>Have you been tested for Covid-19 in the past (PCR test)?<br>☐ Yes I've been tested<br>☐ No<br><br>If yes - Have you been tested positive to COVID-19 in one of the tests?<br><br>1.Yes, on the date XX/XX/XX, Did you repeat the test from then? YES/NO<br>If yes -what was the last date in which you were tested? XX/XX/XXX<br>What was the result of the last test? Positive/Negative/ did not get the result	
Healthcare question	US-CMU/UMD	"In the past 5 days, have you worked or volunteered in a hospital, medical office, ambulance service, first responder services, or any other health care setting?"	

	Ex-US-CMU/UMD	In the last 7 days, did you do any work for pay, or do any kind of business, farming, or other activity to earn money, even if only for one hour? 1=Yes 2=No<br><br>[Respondents who answered Yes were asked] What is the main activity of the business or organization in which you work?<br><br>[Respondents who answered No were asked] Before February 2020, were you working for pay, or doing any kind of business, farming, or other activity to earn money?<br><br>[Respondents who answered Yes were asked] What is the main activity of the business or organization in which you were working before February 2020?<br><br>Healthcare workers were defined as those selecting [7 = Health] to either of the above profession questions.	Question introduced 27 June 2020
	CSS UK	Are you a healthcare worker (including hospital, elderly care or in the community)<br>☐ Yes currently treat patients<br>☐ Yes do not currently treat patients<br>☐ Yes currently interact with patients<br>☐ Yes but do not currently interact with patients<br>☐ No	
	Israel Corona	Do you work in a medical team, actively treating patients?<br>☐ Yes I’m a healthcare professional<br>☐ No	

Supplementary table 2: Weighted analyses versus demographic characteristics for CMU/UMD Facebook COVID-19 Symptom Survey platform in the US, UK and Israel.

Baseline demographic characteristics of non-healthcare worker CMU/UMD platform survey respondents age ≥ 18 years reporting male or female sex, in relation to the census population, with and without application of survey weights.

	United States			United Kingdom			Israel		
	Raw Survey	Survey-Weighted	Country	Raw Survey	Survey-Weighted	Country	Raw Survey	Survey-Weighted	Country
Age (adults only) mean (sd)	48.5 (16.3)	48.7 (0.01)	47.8 (18.3)	43.0 (15.6)	48.3 (0.05)	48.6 (18.6)	47.5 (17.5)	45.4 (0.09)	44.9 (18.5)
Sex (%male)	33.3	48.1	49.5	36.9	49.2	49.5	49.1	47.8	48.8

Supplementary table 3 – For each symptom, overall OR observed for the population combined from the six different sources along with their 95% confidence interval

Symptom	Aggregated OR and 95% CI
anosmia/ageusia	16.96 [13.13 ; 21.92]
fever	6.45 [4.25 ; 9.81]
shortness of breath	4.69 [3.14 ; 7.01]
cough	4.29 [3.13 ; 5.88]
chest pain/pressure	3.57 [2.85 ; 4.48]
fatigue/exhaustion	3.43 [2.49 ; 4.73]
myalgias/arthralgias	3.52 [2.19 ; 5.66]
headache	3.46 [2.76 ; 4.34]
pharyngitis	2.48 [1.71 ; 3.59]
nausea/vomiting	2.39 [1.49 ; 3.82]
diarrhoea	1.86 [1.63 ; 2.12]
